# Effect of dietary inclusion of *Bacillus*-based probiotics on performance, egg quality, and the faecal microbiota of laying hen

**DOI:** 10.5713/ab.23.0299

**Published:** 2024-01-20

**Authors:** Habeeb Tajudeen, Sang Hun Ha, Abdolreza Hosseindoust, Jun Young Mun, Serin Park, SangIn Park, PokSu Choi, Rafael Gustavo Hermes, Apichaya Taechavasonyoo, Raquel Rodriguez, JinSoo Kim

**Affiliations:** 1 Department of Animal Industry Convergence, Kangwon National University, Chuncheon, 24341, Korea; 2 Kemin Industries Inc Headquarters, 1900 Scott Ave Des Moines, Des Moines, IA, 50317, USA

**Keywords:** Commercial Layer, Egg Production, Gastrointestinal Flora, Laying Performance, Microbiota, Mucosa Structure

## Abstract

**Objective:**

Our study examined the impact of propriety blends of *Bacillus* strain probiotics on the performance, egg quality, and faecal microflora of laying hens.

**Methods:**

A total of 183 Institut de selection Animale (ISA) brown laying hens aged 23 weeks with an average body weight of 1,894±72 g were randomly allocated into 3 groups as control (corn-soybean meal based diet, CON), 0.5 g/kg Enterosure probiotics (ET1, 3×10^8^ colony-forming unit [CFU]/kg feed), and 5 g/kg Enterosure probiotics (ET2, 3×10^9^ CFU/kg feed) administered in mashed form. At the completion of each phase hen day egg production (HDEP), average egg weight (AEW), feed intake, and faecal microbiota were evaluated.

**Results:**

HDEP and AEW were higher (p<0.05) in the ET2-supplemented diet in phase 3 (week 9 to 12) compared with CON. Egg mass (EM) was higher (p<0.05) in phase 2 at ET2, and also higher (p<0.05) in phase 3 at the ET1 and ET2-supplemented diets compared with CON. Feed conversion ratio was lower (p<0.05) in phase 3 at the ET1 and ET2-supplemented diets, with ET2 being the lowest compared with ET1 and CON. Yolk colour was higher (p<0.05) in the ET-supplemented diets at phase 3 compared with CON. *Bifidobacterium* spp. was higher (p<0.05) in the ET2-supplemented diet compared with CON in phase 2, while in In phase 3, *Lactobacillus* spp. and *Bifidobacterium* spp. were higher (p<0.05) in the ET-supplemented diets compared with CON. *Coliforms* were lower (p<0.05) in the ET-supplemented diets compared with CON in phase 3.

**Conclusion:**

The propriety blends of *Bacillus* strain probiotics supplements at 0.5 g/kg and 5 g/kg could improve the production and quality of eggs with more significance at 5 g/kg for HDEP, AEW and EM, which was achieved via the increase of beneficial microbiomes such as *Lactobacillus* spp., *Bifidobacterium* spp., and the decrease of pathogenic microbiomes like *Escherichia coli* and *Coliforms* which was speculated to improve gut barrier function and the reproductive hormone.

## INTRODUCTION

The poultry industry is constantly striving to increase output due to market demands, resulting in the widespread use of antibiotics and growth boosters in poultry production [1]. However, the misuse of antibiotics in poultry production has stimulated the emergence of antibacterial resistance and dysbiosis in poultry birds [2], making antimicrobial resistance a global concern [3]. A gradual shift from its use in recent times has led to a number of research projects devoted to the characterization of the gastrointestinal system in regard to bacterial population and functional content, which has improved the knowledge of gut microbiota's significant impact on livestock nutrition and health. The gut microbiota is a colony of various microorganisms which can be improved with the use of probiotic supplements in the diets of poultry birds [4,5]. Probiotics affect the gut by single or several methods, including as a modifier to the host immune system, supplying energy via the generation of short-chain fatty acids, and affecting gut morphology, integrity, and its operation [6]. Probiotics also have a profound influence on harmful bacteria as they produce metabolites and antimicrobial substances, occupying the microhabitats in the gut to actively prevent their colonization by reducing intestinal luminal pH [4].

Chickens supplemented with probiotics in their diet have been reported to have improved egg production and quality, and optimum feed efficiency [7], as a result of an alteration of the gastrointestinal flora in both layers and broilers [8,9]. The majority of the probiotics employed in chicken husbandry originate from the genus *Bifidobacterium*, *Lactobacillus*, *Bacillus*, and *Lactococcus* [10]. However, there is ongoing research on the use of spore-forming *Bacilli* probiotics as a specific strain of bacteria or as mixed species to eliminate the colonization of pathogens [11]. An in-depth understanding of the molecular mode of action will enable proper future use. Therefore, the objective of the present novel study was to investigate the effects of Enterosure probiotics (ET) on performance, egg quality, and faecal microbiota of laying hens. The ET probiotics were produced via the proprietary blend of *Bacillus subtilis* PB6 with strain ID: ATCC PTA-6737, *Bacillus subtilis* FXA with strain ID: ATCC PTA-127113, and *Bacillus licheniformis* G3 with strain ID: ATCC PTA-127114. This blend affects the intestinal health and enhances animal productivity by secreting metabolites that are antibiotic in nature, forming a quorum quenching molecule to specifically hinder the growth of harmful bacteria and promote the healthy microbiomes as its mode of action

## MATERIALS AND METHODS

### Animal care and ethical statement

This experiment was conducted at the facility of Kangwon National University. All the procedures used in this experiment were approved by the Institutional Animal Care and Use Committee of Kangwon National University (KW-220413-1).

### Animal, diets, and experimental design

A total of 189 23-week-old Institut de selection Animale (ISA) brown laying hens (average body weight at the start = 1,894 ±72 g, were randomly divided into 3 groups: control (corn-soybean meal based diet, CON), 0.5 g/kg ET (ET1, 3×10^8^ colony-forming unit [CFU]/kg feed), and 5 g/kg ET (ET2, 3×10^9^ CFU/kg feed), with 7 replicates per group, and 9 hens per replicate. The trial lasted for a total of 14 weeks and was initiated during the summer period for 98 days including a two-weeks adaptation period. The two-week adaptation period (14 d) started when hens were 23 weeks old; during this period all hens received the same diet (without the test product). When hens were 25 weeks old the experimental period started and hens received either the CON or one of the treatment diets for 12 weeks (84 days). The ingredient and calculated main nutrient composition of the basal diet are presented in Table 1. All diets met or exceeded the commercial layer requirements recommended by ISA brown breeder company. Feed and water were provided *ad libitum* and feed was presented in mash form. The experimental diets were provided for 12 weeks in three feeding phases (phase 1, 0 to 4 weeks; phase 2, 5 to 8 weeks; phase 3, 9 to 12 weeks). All hens were kept in a window-less room, with room temperature kept at 20°C to 22°C. The light cycle was 16 h of continuous light and 8 h of the dark period. Temperature and humidity were controlled as ISA brown Management guides by an automatic ventilation system (19°C and 60%, temperature and humidity, respectively). Cages were enriched according to the EU standards; each cage was equipped with two individual nipples, a feed trough (15 cm long; providing 15 cm/hen), a perch, a nest, and a claw-shortening device. Each cage had a total area of 6,400 cm^2^ (providing 1,225 cm^2^/hen). Animals and facility were inspected twice daily (checking for general health status and welfare, constant feed and water supply as well as temperature and ventilation, mortalities, and other unexpected events).

### Diets formulation

Feeds were formulated at the Kangwon National University’s laying hen breeding facility. The feeds were thoroughly mixed using the horizontal feed mixer, 1,200 kg, with motor power 1 hp (KH super 15.H.P). The *Bacillus*-based probiotics ET from the blend of *Bacillus subtilis* PB6 (ATCC PTA-6737) originating from the intestine of healthy birds during a necrotic enteritis outbreak, *Bacillus subtilis* FXA (ATCC PTA-127113) originating from the faeces of chicken and *Bacillus licheniformis* G3 (ATCC PTA-127114) that originated from animal water tanks was supplemented as a premix in a powder form and was added through a continuous mixing method. All formulated feeds were properly bagged, weighed, and kept airtight away from contaminants after formulation.

To avoid cross contamination CON feeds were thoroughly mixed before supplementing feeds with test products. Precautions were taken during the collection and subsequent sample handling that contamination did not occur. To avoid contamination at the farm, hens from each cage only had access to their feeders. Precautions were also taken at farm level to avoid contaminations. In the feed mill, feed bags were identified with the manufacturing date, net weight, bag number and treatment code. Neither antibiotic, nor coccidiostats or growth promotion additives were added to the diets.

### Chemical analysis

The proximate analysis of the feed samples was performed by the Monogastric Animal Nutrition Laboratory, Kangwon National University. Diet was analysed for dry matter as described in 930.15; AOAC [12], crude protein 990.03; AOAC [12], ether extract 2003.03; AOAC [12], ash 942.05; AOAC [12].

### Data and sample collection

#### Laying performance

At the completion of each phase at (4, 8, and 12 weeks), parameters such as feed conversion ratio (kg of feed/kg of eggs, FCR) was evaluated on cage basis via egg production (hen day egg production, HDEP), average egg weight (AEW), and feed intake (FI). Egg production was measured daily and represented as (% hens/d). The AEW weight for our study was determined by evaluating daily egg weights after separating debris, and egg mass (EM) was estimated via the multiplication of AEW and HDEP.

#### Egg and eggshell quality

Tests on the quality of eggs and eggshells such as Haugh units (HU), yolk colour, yolk and albumin percentages, yolk and albumin weights, shell thickness and hardness were evaluated after harvesting eggs at the conclusion of each phase. Egg multi-tester (Tohoku rhythm co., Ltd., Tokyo, Japan) was employed for evaluating HU, yolk and albumin weights, and yolk colour. A type II egg shell force gauge (Robotmation Co., Ltd., Tokyo, Japan) was adopted to measure the eggshell's strength value. The nature of eggshell thickness was evaluated by a dial pipe gauge (Ozaki MFG. Co., Ltd., Tokyo, Japan) which was specifically based on the thickness of the round edge, sharp edge, and the midsection of the egg while excluding the interior membrane.

#### Faecal microbiota DNA extraction

Fresh faecal samples were obtained from seven hens per treatment at the conclusion of each phase via the cloaca by gentle palpation to stimulate the excretion of fresh faeces into airtight tubes to circumvent external contaminations. The samples were then preserved immediately at −80°C until analysis in order to measure the differences in faecal microbiota according to each treatment. It was done in accordance with the QIAamp fast DNA stool mini kit Germany cat. No. 51604 2016 extraction methodology as previously used by Tajudeen et al [13].

#### Real time polymerase chain reaction (QPCR)

The quantitative polymerase chain reaction (qPCR) employed for quantifying *Lactobacillus* spp., *Bifidobacterium* spp., *Clostridium* spp., *Enterococcus* spp., *Escherichia coli* (*E. coli*), and *Coliforms* in our study was carried out subsequently after the extraction process stated above. In detail; 10 μL of the extracted deoxyribonucleic acid (DNA), 2.5 ng/μL of forward and backward primers, and 1× universal SsoAdvanced universal SYBR Green Supermix were administered. A reaction containing 10 ng of DNA, 2.5 ng/μL of forward and reverse primers, and 1× universal SsoAdvanced universal SYBR Green Supermix were added [14,15]. The SYBR thermal cycling protocol and primers used are described in Table 2 with beta actin (β-actin) as the housekeeping gene. Enzymatic activation was attained at the cycling parameters of 95°C then 40 cycles of melting at 95°C for 15 s; annealing for a set times and temperatures in accordance with individual primer details (Bioneer Corporation, Daejeon, Korea); and extension at 72°C for a set period according to each primer. Identified bacterial species were thoroughly mixed 10-fold before being employed to generate PCR findings while the SYBR green fluorescence signals were being monitored at 72°C. The DNA amplification was performed using the qPCR Rotor-Gene Qiagen 2plex software with Serial Number 0312272, developed by Corbett Research Life Science Qiagen 2008 [13].

### Statistical analysis

The statistical analysis system (SAS, 2012) was applied to run the general linear model approach in a randomised complete block design on the dataset provided for the current study. The Tukey's honest significant difference test was used to distinguish between significant differences between treatment means. The experimental unit consisted of replicate cages accommodating seven laying hens each, and probability values of p<0.05 were considered significant [16].

## RESULTS

### Laying performance

The results show that HDEP was not significant in phase 1 (week 0 to 4) across all treatments (Table 3). There was a tendency (p = 0.067) towards significance in phase 2 (week 5 to 8) for HDEP at the ET-supplemented diets compared with CON. Phase 3 (week 9 to 12) showed higher (p<0.05) HDEP in the ET2-supplemented diets compared with CON. AEW was not significant in phase 1 and phase 2 across all treatments. However, the AEW was higher (p<0.05) in phase 3 at the ET2-supplemented diets compared with CON. EM was not significant in phase 1 of our experiment across all treatments, but it was higher (p<0.05) in phase 2 at ET2, and also higher (p<0.05) in phase 3 at the ET1 and ET2-supplemented diets compared with CON. FCR was not significant in phase 1 and 2 of our experiment. However, it was lower (p<0.05) in phase 3 at the ET1 and ET2-supplemented diets, with ET2 being the lowest compared with ET1 and CON. There were no differences in BW and average daily feed intake.

### Egg and eggshell quality

There was no significant difference in yolk colour at phase 1 and phase 2. However, yolk colour was higher (p<0.05) in the ET-supplemented diets at phase 3 compared with CON (Table 4). There was no significant difference in HU, yolk weight, albumin weight, yolk percentage, and albumin percentage across all treatments and phases. While in Table 5, there were no significant differences among different treatments in eggshell thickness and hardness for any of the periods of the study.

### Faecal microbiota

**Figure 1:**
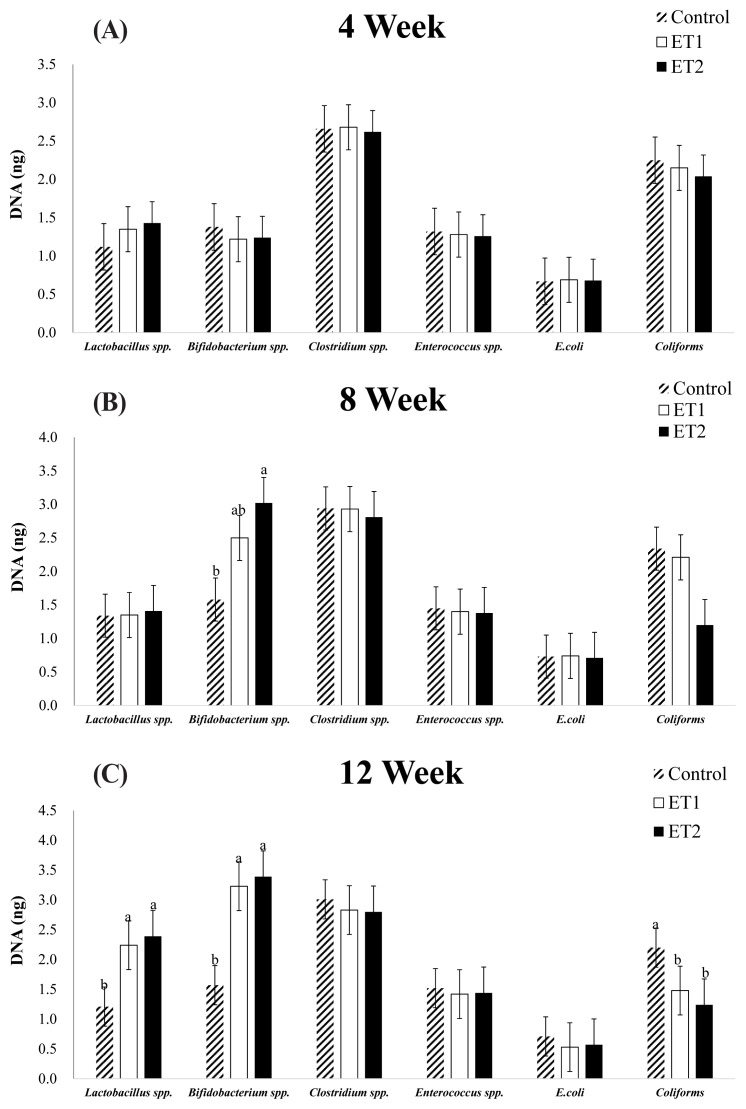
Effects of *bacillus*-based probiotic supplementation on faecal microflora DNA of laying hens. (A) 4 week, (B) 8 week, (C) 12 week. Error here represents standard error of means. Bars With different letters differ significantly across all treatments. ^1)^ CON, basal diet; ET1, 0.5 g/kg probiotics + basal diet; ET2, 5 g/kg probiotics + basal diet. ^a,b^ The same letter indicates no significant difference, whereas different letter indicates significant differences (p<0.05).

In Figure 1, there were no significant differences in phase 1 (week 4; Figure 1a) and 2 (week 8; Figure 1b) for all the faecal microbiota measured, except for *Bifidobacterium* spp. which was higher (p<0.05) in the ET2-supplemented diet compared with CON (week 8; Figure 1b). In phase 3 (week 12; Figure 1c), *Lactobacillus* spp. and *Bifidobacterium* spp. were higher (p<0.05) in the ET-supplemented diets compared with CON. *Coliforms* were lower (p<0.05) in the ET-supplemented diets compared with CON. A tendency was reported for *E. coli* in phase 3 (p = 0.052) with a numerical decrease in ET1 and ET2 compared with CON. There were no significant differences in *Clostridium* spp. and *Enterococcus* spp. among the treatments across all phases.

## DISCUSSION

In our study, the FCR was significantly improved in the ET-supplemented diets. The ability of *Bacillus* to produce digestive-aiding enzymes such as proteases, amylases and cellulases could explain the improvement in feed utilisation and conversion [17]. The reduced FCR reported in the ET-supplemented groups indicates higher efficiency, and it is in agreement with other authors who have reported similar results on FCR after dietary supplementation with *Bacillus subtilis* [18,19]. It has been described that probiotic enhances the gut mucosa's structure [20], which typically improves the integrity of the epithelial barrier by forming a stable barrier resistant to pathogens such as *E. coli* and *Coliforms* as shown in our study. This is achieved by strengthening the dominance of tight junction proteins that adhere to each other thereby forming a stable barrier resistant to pathogens and larger molecules [21]. We propose from our findings that ET which is a *Bacillus* strain probiotics activated its mode of action for optimum FCR by improving intestinal mucosa structure and gut health.

A modulation of faecal microbiota in our study could explain the improved laying performance such as increased HDEP, AEW, and EM observed in the ET-supplemented groups. This is in agreement to the study of Abd et al [22] where *Bacillus* probiotics improved the reproductive performance of layer hens. They proposed the characteristics might be driven by the *Bacillus* variant producing abundant metabolites and lytic enzymes exhibiting anti-oxidative and DNA-protective capabilities. *Bacillus* is effective in synthesising digestive aiding enzymes, and reduction in the multiplication of pathogenic bacteria [23]. In addition, there has been a report of direct relationships between the gut microbiome and the reproductive system [24]. In their report, it was stated that oestrogen level is primarily regulated by the gut microbiome, and the gene repertoire of the gut microbiota that stimulates oestrogen metabolism is referred to as the "oestrobolome”. Although this was not measured in our study, the origin and modulation of sexual differentiation in avian requires the activation of several hormones that are fundamentally influenced by the oestrogen levels primarily regulated by positive gut microbiomes such as *Lactobacillus* [25]. They further explained that *Bacillus* strain of probiotics improved the production and quality of eggs by strengthening gut barrier function, upregulating oestrogen receptor hormone, follicle-stimulating hormone, and sex steroids known as oestradiol. We therefore speculate that the increase in laying performance as observed in this study might be as a result of an increase in the prevalence of *Lactobacillus* spp., *Bifidobacterium* spp., and a decrease in *E. coli* and *Coliforms* in the supplemented diets, being that the level of microbiota in the gut of a laying hen is proportional to the performance and health status.

Egg yolk colour is an important marker of preference for consumers in many countries. This is due to the fact that a golden yellow colour is preferred to a pale-yellow colour. However, laying hens lack the ability to individually synthesise yolk colour except via supplementations [26]. In our experiment, Yolk colour was higher in the ET-supplemented diets compared to CON. This shares similarity with the study of Sobczak and Kozlowski [27] which states that *Bacillus* supplemented diets had higher significant score for egg yolk. It can be predicted that the absorption and proper transfer of dietary carotenoids was achieved by *Bacillus* probiotics supplementation due to a better gut health and nutrient’s absorption. Diets high in carotenoids are linked to reduced cholesterol levels with possible increase in xanthophyll and the level of β-carotene [28,29]. Poultry feeds generally contains numerous sources of available pigments such as maize as shown in our experimental diet containing sufficient amount of corn. However, the pigmenting efficacy is determined by factors such as digestion, accumulation of carotenoids in the target tissue, and the gut health [29]. Thus, we propose that the better yolk colour in the third phase our study can be linked to the better gut health of laying hen as described by the quality of microbiota in our study.

The practical implications of our finding for the poultry industry is that it will help curb the reliance on antibiotics and growth promoters for improving gut health, microbiota quality, and laying performance in chicken. This aligns with the growing trend to reduce the use of antibiotics due to concerns about antimicrobial resistance, environmental antibiotic residue, and consumer preferences.

## CONCLUSION

We propose from our results that ET-supplemented diets at level 5 g/kg (3×10^9^ CFU/kg feed) could yield improvement in laying performance, AEW, and EM by increasing the beneficial microbiomes such as *Lactobacillus* spp., *Bifidobacterium* spp., tentatively decreasing pathogenic microbiomes like *E. coli* and *Coliforms*.

## Figures and Tables

**Table 1:** Ingredient and calculated composition of basal diet

Items	CON
Ingredient (%)	
Corn	62.20
Wheat bran	1.53
Soybean meal	24.00
Animal fat	1.50
Limestone	8.55
Tricalcium phosphate	1.40
Vitamin and mineral premix^1)^	0.32
Sodium chloride	0.31
DL-methionine, 50%	0.19
Total	100
Calculated and determined composition (g/kg)	
Metabolizable energy (kcal/kg)	2,750
Crude protein	16.00
Dry matter	91.45
Ash	9.95
Crude fibre	4.13
Crude fat	3.95
Ca	3.50
P	0.80
Lys dig	0.84
Met dig	0.41
Met-Cys dig	0.66

1) Provides per kilogram of diet: Vit A (retinyl acetate) 10,000 IU; Vit D_3_ (cholecalciferol) 2,000 IU; Vit E (dl-a-tocopherol) 0.25 IU; Vit K_3_ (menadione) 2 mg; Vit B_12_ (cyanocobalamin) 10 mg; Choline chloride 250 mg; Folic acid 1 mg; Niacin 30 mg; D-pantothenic acid 10 mg; Vit B_6_ (pyridoxine-HCl) 3 mg; Vit B_2_ (riboflavin) 6 mg; Vit B_1_ (thiamin) 2 mg; Antioxidant (ethoxyquin) 125 mg; Cobalt 0.3 mg; Copper 10 mg; Iron 60 mg; Iodine 0.5; Manganese 40 mg; Selenium 0.2 mg; Zinc, 50 mg.

**Table 2:** Cycling details of primers used in this study

Microflora	Primer sequence	Anneal/extension temperature	Cycles
*Lactobacillus* spp.	F: DNA-AGC AGT AGG GAA TCT TCC A	54	40
	R: DNA-CAC CGC TAC ACA TGG AG	53.6	
*Bifidobacterium* spp.	F: DNA-TCG CGT CYG GTG TGA AAG	59.4	
	R: DNA-CCA CAT CCA GCR TCC AC	55.9	
*Clostridium* spp.	F: DNA-GGC GGC YTR CTG GGC TTT	62.1	
	R: DNA-CCA GGT GGA TWA CTT ATT GTG TTA A	56.1	
*Enterococcus* spp.	F: DNA-CCC TTA TTG TTA GTT GCC ATC ATT	50	
	R: DNA-ACT CGT TGT ACT TCC CAT TGT	50	
*E. Coli*	F: DNA-AAA ACG GCA AGA AAA AGC AG	55	
	R: DNA-GCG TGG TTA CAG TCT TGC G	58.6	
*Coliform*	F: DNA-TGA TTT CCG TGC GTC TGA ATG	55	
	R: DNA-ATG CTG CCG TAG CGT GTT TC	58	
β-Actin	F: DNA-CTC CTT CCT GGG CAT GGA	57.3	
	R: DNA-CGC ACT TCA TGA TCG AGT TGA	57.8	

**Table 3:** Effects of *bacillus*-based probiotic supplementation on laying performance

Items	Treatments^1)^			SEM	p-value

	CON	ET1	ET2		
BW (kg)					
4 wk	1.90	1.92	1.86	0.017	0.346
8 wk	1.98	2.03	2.04	0.012	0.110
12 wk	2.02	2.04	2.06	0.019	0.149
ADFI (g/d/bird)					
4 wk	112.84	112..2	111.18	3.181	0.866
8 wk	113.00	113.08	112.69	2.277	0.984
12 wk	113.64	112.80	112.15	1.382	0.569
HDEP (%)					
4 wk	92.85	93.16	93.40	0.585	0.645
8 wk	92.92	93.30	93.94	0.407	0.067
12 wk	92.74^b^	93.61^ab^	94.37^a^	0.435	0.006
AEW (g)					
4 wk	57.61	58.67	58.95	1.085	0.443
5 to 8 wk	58.69	59.26	59.93	0.584	0.134
9 to 12 wk	59.12^b^	60.30^ab^	61.05^a^	0.620	0.019
Egg mass (g/ hen per d)					
0 to 4 wk	53.47	54.66	55.06	0.994	0.278
5 to 8 wk	54.53^b^	55.30^ab^	56.36^a^	0.631	0.032
9 to 12 wk	54.83^b^	56.45^a^	57.61^a^	0.578	<0.001
FCR					
0 to 4 wk	2.113	2.057	2.021	0.067	0.409
5 to 8 wk	2.073	2.046	2.000	0.049	0.344
9 to 12 wk	2.086^a^	1.999^b^	1.912^c^	0.024	<0.001

SEM, standard error of means; BW, body weight; ADFI, average daily feed intake; HDEP, hen day egg production; AEW, average egg weight; FCR, feed conversion ratio.

1) CON, basal diet; ET1, 0.5 g/kg probiotics + basal diet; ET2, 5 g/kg probiotics + basal diet.

a–c The same superscript indicates no significant difference, whereas different superscript indicates significant differences (p<0.05).

**Table 4:** Effects of *bacillus*-based probiotic supplementation on egg qualities

Items	Treatments^1)^			SEM	p-value

	CON	ET1	ET2		
Haugh units					
4 wk	90.06	90.87	91.51	0.697	0.715
8 wk	88.23	90.03	90.29	1.062	0.711
12 wk	85.46	87.44	88.03	0.827	0.434
Yolk colour					
4 wk	7.21	7.53	7.67	0.162	0.521
8 wk	7.00	7.43	7.54	0.111	0.109
12 wk	6.78^b^	7.19^a^	7.31^a^	0.092	0.035
Yolk weight (g)					
4 wk	13.06	13.67	13.77	0.139	0.075
8 wk	13.92	14.38	14.48	0.125	0.148
12 wk	14.20	14.69	15.11	0.187	0.134
Albumin weight (g)					
4 wk	38.74	39.35	39.75	0.429	0.651
8 wk	39.39	39.40	39.75	0.199	0.720
12 wk	39.40	39.89	40.26	0.293	0.505
Yolk percentage (%)					
4 wk	22.66	23.31	23.36	0.178	0.218
8 wk	23.72	24.24	24.16	0.180	0.442
12 wk	24.02	24.36	24.75	0.290	0.610
Albumin percentage (%)					
4 wk	67.22	67.03	67.41	0.352	0.918
8 wk	67.12	66.48	66.34	0.230	0.355
12 wk	66.64	66.14	65.95	0.327	0.697

SEM, standard error of means.

1) CON, basal diet; ET1, 0.5 g/kg probiotics + basal diet; ET2, 5 g/kg probiotics + basal diet.

a,b The same superscript indicates no significant difference, whereas different superscript indicates significant differences (p<0.05).

**Table 5:** Effects of *bacillus*-based probiotic supplementation on eggshell qualities

Items	Treatments^1)^			SEM	p-value

	CON	ET1	ET2		
Eggshell thickness (mm)					
4 wk	0.40	0.42	0.43	0.024	0.440
8 wk	0.40	0.43	0.42	0.020	0.492
12 wk	0.42	0.41	0.40	0.022	0.712
Eggshell hardness					
4 wk	4.50	4.59	4.53	0.252	0.942
8 wk	4.77	4.81	4.79	0.115	0.931
12 wk	4.86	4.80	4.93	0.163	0.735

SEM, standard error of means.

1) CON, basal diet; ET1, 0.5 g/kg probiotics + basal diet; ET2, 5 g/kg probiotics + basal diet.

## References

[1] Diarra MS, Francois M (2014). Antibiotics in Canadian poultry productions and anticipated alternatives. Front Microbiol.

[2] Stanton TB (2013). A call for antibiotic alternatives research. Trends Microbiol.

[3] WHO (2018). Antimicrobial resistance.

[4] Khan S, Moore RJ, Stanley D, Chousalkar KK (2020). The gut microbiota of laying hens and its manipulation with prebiotics and probiotics to enhance gut health and food safety. J Appl Environ Microbiol.

[5] Tsai MY, Shih BL, Liaw RB (2023). Effect of dietary supplementation of Bacillus subtilis TLRI 211-1 on laying performance, egg quality and blood characteristics of Leghorn layers. Anim Biosci.

[6] Aalaei M, Khatibjoo A, Zaghari M, Taherpour K, Gharaei M, Soltani M (2018). Comparison of single- and multi-strain probiotics effects on broiler breeder performance, egg production, egg quality and hatchability. Br Poult Sci.

[7] Lokapirnasari WP, Pribadi TB, Al Arif A (2019). Potency of probiotics Bifidobacterium spp. and Lactobacillus casei to improve growth performance and business analysis in organic laying hens. Vet World.

[8] Koenen ME, Kramer J, Van Der Hulst R, Heres L, Jeurissen SHM, Boersma WJA (2004). Immunomodulation by probiotic lactobacilli in layer- and meat-type chickens. Br Poult Sci.

[9] Yu B, Liu JR, Hsiao FS, Chiou PWS (2008). Evaluation of Lactobacillus reuteri Pg4 strain expressing heterologous β-glucanase as a probiotic in poultry diets based on barley. Anim Feed Sci Technol.

[10] Jha R, Das R, Oak S, Mishra P (2020). Probiotics (direct-fed microbials) in poultry nutrition and their effects on nutrient utilization, growth and laying performance, and gut health: a systematic review. Animals.

[11] Hosseindoust A, Mohammadi M, Yao ZP, Jung M, Kim IH (2018). Dietary Bacillus subtilis B2A strain in laying hens challenged with Salmonella gallinarum: effects on egg production, egg quality, blood haptoglobin and targeted intestinal Salmonella shedding. J Appl Anim Res.

[12] AOAC International (2007). Official methods of analysis of AOAC International.

[13] Tajudeen H, Mun JY, Ha SH, Hosseindoust A, Lee S, Kim JS (2023). Effect of wild ginseng on the laying performance, egg quality, cytokine expression, ginsenoside concentration, and microflora quantity of laying hens. J Anim Sci Technol.

[14] Stringer AM, Gibson RJ, Logan RM, Bowen JM, Yeoh AS, Keefe DM (2008). Faecal microflora and β-glucuronidase expression are altered in an irinotecan-induced diarrhea model in rats. Cancer Biol Ther.

[15] Muhammad A, Mohamed DA, Chwen LT, Akit H, Samsudin AA (2021). Effect of selenium sources on laying performance, egg quality characteristics, intestinal morphology, microbial population and digesta volatile fatty acids in laying hens. Animals.

[16] SAS (2012). Statistical Analysis Software for PC. Release 9. 3.

[17] Mazanko MS, Gorlov IF, Prazdnova EV (2018). Bacillus probiotic supplementations improve laying performance, egg quality, hatching of laying hens, and sperm quality of roosters. Probiotics Antimicrob Proteins.

[18] Guo JR, Dong XF, Liu S, Tong JM (2017). Effects of long-term Bacillus subtilis CGMCC 1.921 supplementation on performance, egg quality, and fecal and cecal microbiota of laying hens. Poult Sci.

[19] Molnar AK, Podmaniczky B, Kurti P (2011). Effect of different concentrations of Bacillus subtilis on growth performance, carcase quality, gut microflora and immune response of broiler chickens. Br Poult Sci.

[20] Abdelqader A, Al-Fataftah AR, Das G (2013). Effects of dietary Bacillus subtilis and inulin supplementation on performance, eggshell quality, intestinal morphology and microflora composition of laying hens in the late phase of production. Anim Feed Sci Technol.

[21] Chichlowski M, Croom J, McBride BW, Havenstein GB, Koci MD (2007). Metabolic and physiological impact of probiotics or direct-fed-microbials on poultry: a brief review of current knowledge. Int J Poult Sci.

[22] Abd El-Hack ME, Mahgoub SA, Alagawany M, Ashour EA (2017). Improving productive performance and mitigating harmful emissions from laying hen excreta via feeding on graded levels of corn DDGS with or without Bacillus subtilis probiotic. J Anim Physiol Anim Nutr.

[23] Wealleans AL, Sirukhi M, Egorov IA (2017). Performance, gut morphology and microbiology effects of a Bacillus probiotic, avilamycin and their combination in mixed grain broiler diets. Br Poult Sci.

[24] Qi X, Yun C, Pang Y, Qiao J (2021). The impact of the gut microbiota on the reproductive and metabolic endocrine system. Gut Microbes.

[25] Wang Y, Du W, Lei K (2017). Effects of dietary Bacillus licheniformis on gut physical barrier, immunity, and reproductive hormones of laying hens. Probiotics Antimicrob Proteins.

[26] Lokaewmanee K, Yamauchi K, Komori T, Saito K (2011). Enhancement of egg yolk color by paprika combined with a probiotic. J Appl Poult Res.

[27] Sobczak A, Kozlowski K (2015). The effect of a probiotic preparation containing Bacillus subtilis ATCC PTA-6737 on egg production and physiological parameters of laying hens. Ann Anim Sci.

[28] Yeum KJ, Russell RM (2002). Carotenoid bioavailability and bioconversion. Annu Rev Nutr.

[29] Yuniarti M, Bidura IG, Siti NW (2023). Effect of probiotic supplementation in the diet on the production and physical quality of eggs in laying hens. World J Biol Pharm Health Sci.

